# The Histone Deacetylase Inhibitor, Vorinostat, Represses Hypoxia Inducible Factor 1 Alpha Expression through Translational Inhibition

**DOI:** 10.1371/journal.pone.0106224

**Published:** 2014-08-28

**Authors:** Darren M. Hutt, Daniela Martino Roth, Hélène Vignaud, Christophe Cullin, Marion Bouchecareilh

**Affiliations:** 1 Department of Cell and Molecular Biology, The Scripps Research Institute, La Jolla, California, United States of America; 2 Institut de Biochimie et Génétique Cellulaires, CNRS UMR 5095, Université de Bordeaux, Bordeaux, France; University of Dundee, United Kingdom

## Abstract

Hypoxia inducible factor 1α (HIF-1α) is a master regulator of tumor angiogenesis being one of the major targets for cancer therapy. Previous studies have shown that Histone Deacetylase Inhibitors (HDACi) block tumor angiogenesis through the inhibition of HIF-1α expression. As such, Vorinostat (Suberoylanilide Hydroxamic Acid/SAHA) and Romidepsin, two HDACis, were recently approved by the Food and Drug Administration (FDA) for the treatment of cutaneous T cell lymphoma. Although HDACis have been shown to affect HIF-1α expression by modulating its interactions with the Hsp70/Hsp90 chaperone axis or its acetylation status, the molecular mechanisms by which HDACis inhibit HIF-1α expression need to be further characterized. Here, we report that the FDA-approved HDACi Vorinostat/SAHA inhibits HIF-1α expression in liver cancer-derived cell lines, by a new mechanism independent of p53, prolyl-hydroxylases, autophagy and proteasome degradation. We found that SAHA or silencing of HDAC9 mechanism of action is due to inhibition of HIF-1α translation, which in turn, is mediated by the eukaryotic translation initiation factor - eIF3G. We also highlighted that HIF-1α translation is dramatically inhibited when SAHA is combined with eIF3H silencing. Taken together, we show that HDAC activity regulates HIF-1α translation, with HDACis such as SAHA representing a potential novel approach for the treatment of hepatocellular carcinoma.

## Introduction

Hepatocellular carcinoma (HCC) is the most common of all primary liver tumors and represents the third leading cause of cancer related death worldwide [1], [2]. One of the most critical risk factors is cirrhosis, with 90% of HCC cases occurring in cirrhotic livers [3]–[5]. HCC has been linked to dysregulation of diverse signaling pathways affecting cell proliferation, invasion, metastasis and angiogenesis, thereby limiting the development of therapeutic strategies. Currently, the multi-kinase inhibitor Sorafenib is the only treatment approved by the Food and Drug Administration (FDA) for patients with advanced disease, but resection and transplantation remain the only curative treatment available, therefore highlighting the need for identifying novel therapeutic targets [6]–[8].

Histone deacetylase inhibitors (HDACi) are some of the most promising anti-cancer drugs currently being developed [9] with Vorinostat (Suberoylanilide Hydroxamic Acid (SAHA)) and Romidepsin, being recently approved for the treatment of cutaneous T cell lymphoma (CTCL) [10]. A recent study has provided evidence for the therapeutic benefit of HDACi in the treatment of HCC, where the pan-HDACi, panobinostat, improved the efficacy of Sorafenib, resulting in a significant decrease in tumor volume and vessel density, leading to increased survival in HCC xenografts [11]. Furthermore, a phase I clinical trial is underway for the combination treatment of Vorinostat and Sorafenib for patients with advanced renal cell carcinoma and non-small cell lung cancer [12], supporting the use of such combinatorial therapies. Recent evidence has shown that Vorinostat can modulate the IGF-IR signaling pathway and IGF-I promoter activity in endometrial type-I and –II cancers [13], however, the pro-apoptotic activity of this HDACi proved insensitive to a blocking anti-IGF-IR monoclonal antibody. Despite numerous such studies, which provide insight into Vorinostat-responsive signaling pathways, the mechanism of action for the therapeutic benefit of Vorinostat and other HDACi in the treatment of various cancers remains elusive.

Histone deacetylases mediate the removal of acetyl groups from target proteins, which include histones, transcription factors and other cellular proteins, thereby regulating their function. Therefore, HDACi treatment would results in the hyperacetylation of histones and other proteins such as the chaperone Hsp90 [14], [15] involved in regulating the expression and stability of several genes, including those involved in cell-cycle arrest, death and/or apoptosis of cancer cells. In support of this proposed mechanism of action, HDACi have been shown to negatively regulate the expression and function of VEGF, p53 and Hypoxia Inductible Factor-1α (HIF1α), angiogenic factors that promote tumor proliferation and metastasis [16], [17].

HIF-1 is a heterodimeric complex composed of the HIF-1α and HIF-1β subunits and a central regulator of angiogenesis and energy metabolism in tumors [18]–[20]. HIF-1α is regulated by oxygen levels, thereby providing a means of regulating the transcriptional activity of HIF-1 [21]. Under normoxic conditions, proline residues in HIF-1α are hydroxylated by prolyl hydroxylase domain (PHD) containing oxygenases, which serve as a recognition signal for the E3 ubiquitin ligase, Von Hipple-Lindau (VHL) complex, which targets HIF-1α for degradation by the ubiquitin proteosomal system (UPS). Conversely, under hypoxic conditions, the oxygen-dependent PHD-containing oxygenases have reduced activity [22], resulting in stabilization of HIF-1α [23], [24], which dimerizes with the constitutively expressed HIF-1β to activate the transcription of genes involved in angiogenesis, cell proliferation and survival [18], [21], [24]. Cellular HIF-1α levels are also regulated by p53, which promotes MDM2-mediated ubiquitination [21], [25], [26], as well as by autophagy [21], [25], [26]. HIF-1α can also be activated under normoxic conditions by growth factors, oncogenic mutation or inactivation of tumor suppressors [27]–[29]. It is overexpressed in many different types of tumors and mediates resistance to chemo and radiation therapy [20], [21], [30]–[32], making it a primary target for cancer therapy [18], [19].

Previous studies have shown that the HDACi, Trichostatin A (TSA), reduces cellular levels of HIF-1α in a UPS-dependent manner [33]. In support of this observation, Kong *et al* have shown that the silencing of HDAC6 disrupts the Hsp90-mediated folding of HIF-1α, leading to its degradation by the UPS [33]. Conversely, the silencing of HDAC4, which also promotes a down-regulation of this pro-angiogenic factor is dependent on N-terminal lysine residues of HIF-1α [34], suggesting a direct acetylation as a possible regulatory mechanism for HIF-1α. These data suggest that there are multiple HDAC-regulated pathways by which to regulate HIF-1α, suggesting that more evidence is required to better understand the mechanism of action mediating the therapeutic benefit of these HDACi.

Herein, we present evidence that SAHA drastically decreases HIF-1α expression in HCC cell lines by a pathway independent of p53- or prolyl-hydroxylases/VHL-mediated proteasomal degradation and autophagy. Rather, SAHA acts through HDAC9 to alter HIF-1α translation in an eIF3G-dependent manner. We also observed that HIF-1α translation is dramatically inhibited when SAHA is combined with eIF3H silencing. These data provide insight into the mechanism of action of the FDA-approved HDACi, SAHA, which has led us to identify HDAC9, eIF3G and eIF3H as new targets for therapeutic development in hepatocellular carcinoma and possibly other cancers.

## Results

### SAHA down regulates HIF-1α protein levels independent of protein degradation

**Figure 1:**
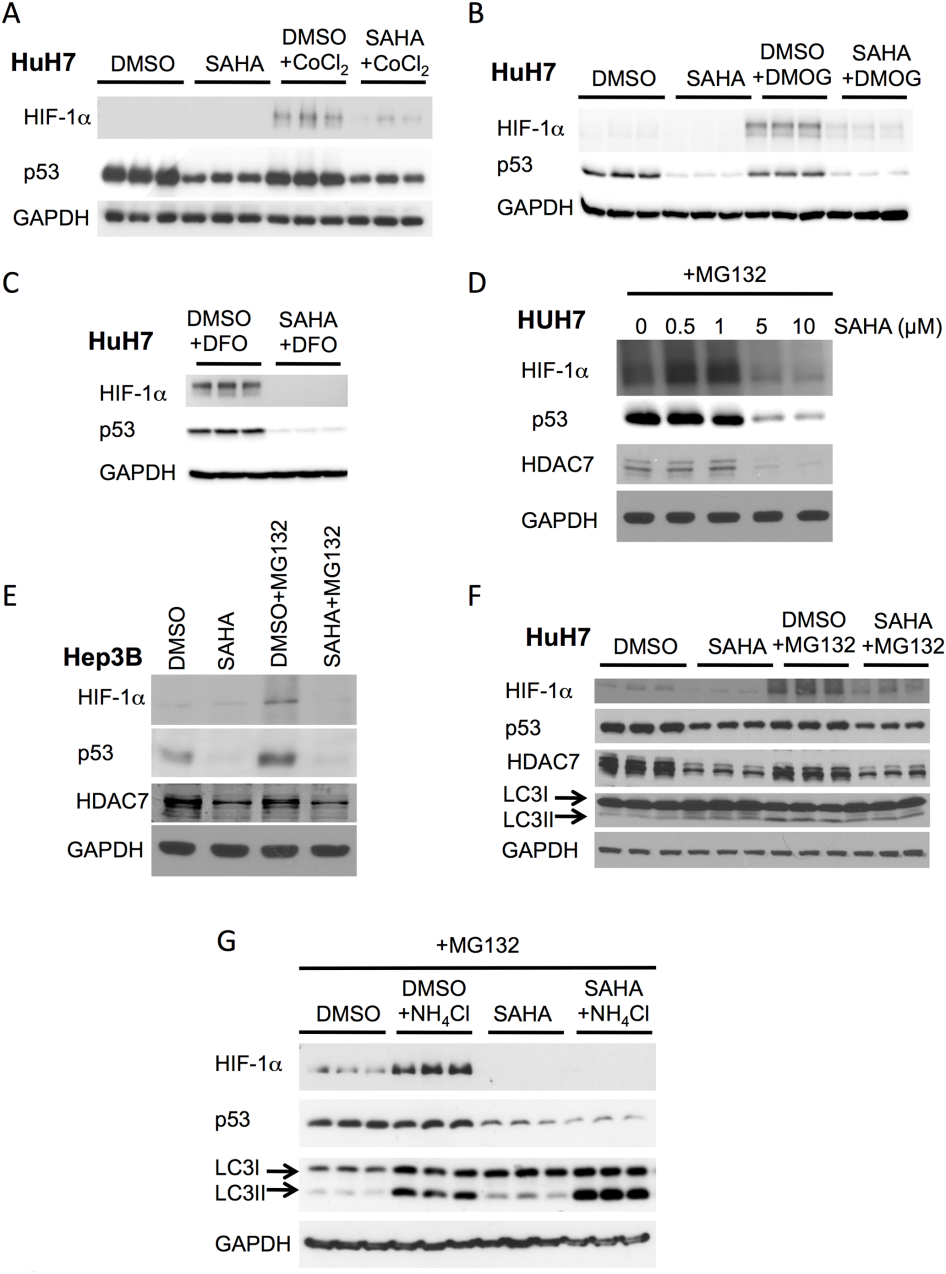
SAHA represses HIF-1α induction in response to hypoxic mimics. (**A**) Immunoblot analysis of HIF-1α, p53 and GAPDH protein expression from cell lysates following treatment of HuH7 cells with 5 µM SAHA, DMSO, DMSO+150 µM cobalt chloride (CoCl_2_) or SAHA+150 µM cobalt chloride (CoCl_2_) for 24 h. (**B**) Immunoblot analysis of HIF-1α, p53 and GAPDH protein expression from cell lysates following treatment of HuH7 cells with 5 µM SAHA, DMSO, DMSO+500 µM dimethyloxallyl glycine (DMOG) or SAHA+500 µM dimethyloxallyl glycine (DMOG) for 24 h. (**C**) Immunoblot analysis of HIF-1α, p53 and GAPDH protein expression from cell lysates following treatment of HuH7 cells with 5 µM SAHA or DMSO for 24 h in the presence or absence of 100 µM desferrioxamine (DFO) for 18 h. (**D**) Immunoblot analysis of HIF-1α, p53, HDAC7 and GAPDH protein expression from cell lysates following treatment of HuH7 cells after SAHA treatment at the indicate concentration is shown in combination with 50 µM MG132 for 4 h. (**E**) and Hep3B cells with 5 µM SAHA or DMSO for 24 h in the presence or absence of 50 µM MG132 for 4 h. (**F**) Immunoblot analysis of HIF-1α, p53, HDAC7 and GAPDH protein expression as well as the splicing of LC3 following treatment of HuH7 cells with 5 µM SAHA or DMSO for 24 h in the presence or absence of 50 µM MG132 for 4 h. (**G**) Immunoblot analyses of HIF-1α, p53 as well as the splicing of LC3 following treatment of HuH7 cells with 50 µM MG132+5 µM SAHA or DMSO in presence or absence of 10 mM ammonium chloride (NH_4_Cl) for 8 h. In all panels GAPDH is used as loading control and HDAC7 is used as control to SAHA treatment.

HDAC inhibitors have garnered a lot of attention in recent years for their therapeutic benefit in treating cancer, with Romidepsin (FK-228) and Vorinostat (SAHA) having been FDA-approved for the treatment of CTCL [10]. In light of the recent clinical trial combining SAHA with Sorafenib for the treatment of patients with advanced cancers [12], we focused our attention on defining the mechanism of action of this HDACi. We first examined the effect of SAHA on HIF-1α protein expression in HuH7 and Hep3B cells, commonly used HCC cell culture models. As mentioned above, HIF-1α protein levels are inversely correlated with the activity of prolyl hydroxylases and therefore the treatment of cells with prolyl hydroxylase inhibitors, such as cobalt chloride (CoCl_2_), desferrioxamine (DFO) or dimethyloxallyl glycine (DMOG) lead to a stabilization in the cellular levels of HIF-1α (Figure 1A–C). We observed that this stabilization in HIF-1α could be abrogated by co-treating cells with SAHA (Figure 1A–C), a result consistent with previously published data showing that HDACi can mitigate the increased expression of this angiogenic factor [33]. We next sought to determine the optimal concentration of SAHA required for reducing HIF-1α protein levels. Here, HuH7 cells were treated with increasing concentrations of SAHA for 24 h and then analyzed for HIF-1α expression. We found that HIF-1α protein levels in HuH7 cells were maximally decreased at a dose of 5 µM (Figure 1D), which coincided with a significant decrease of HDAC7 expression, a result consistent with previous reports on the effect of SAHA on HDAC7 expression [35], [36] (Figure 1D).

To investigate the mechanism underlying the SAHA-mediated repression of HIF-1α protein expression, we used the proteosome inhibitor, MG132, to determine if the UPS was contributing to the SAHA effect, as previously reported for the HDACi, TSA [33]. The treatment of HuH7 and Hep3B cells with MG132 resulted in increased cellular levels of HIF-1α, similar to the effect seen with CoCl_2_, DFO and DMOG, but it failed to block the SAHA effect on decreasing HIF-1α expression (Figure 1E–F), suggesting that the SAHA-mediated reduction in HIF-1α is not occurring by promoting increased proteosomal degradation. We also observed a reduction in p53 levels in response to SAHA treatment of HuH7 and Hep3B cell lines (Figure 1E–F), a result consistent with previously published observations [37]. This reduction was observed whether SAHA was used alone or in combination with CoCl_2_, DFO, DMOG or MG132 (Figure 1A–F). This reduction in p53 protein expression in response to SAHA would have been predicted to reduce the MDM2-dependent ubiquitination of HIF-1α, thereby increasing its expression level and/or activity in HCC cells. These data therefore further support the conclusion that the SAHA-mediated reduction in HIF-1α acts independently of p53 and does not occur by promoting its proteosomal degradation.

In addition to the UPS degradation to clear HIF-1α, the lysosomal pathway has also been described as a clearance pathway for HIF-1α [26]. Therefore to address the contribution of autophagy in the SAHA-mediated reduction in cellular HIF-1α, we monitored the cleavage of LC3-I to its lipidated LC3-II state, a marker of autophagy induction. We did not observe any changes in LC3-I cleavage in response to SAHA treatment, either alone or in combination with MG132 (Figure 1F), suggesting that the SAHA-mediated reduction in cellular HIF-1α levels is not acting through activation of the autophagic pathway. This result was confirmed by our observation that ammonium chloride-mediated inhibition of the lysosome, which alone mediates an increase in HIF1α levels (Figure 1G), failed to block the SAHA-mediated decrease in HIF-1α protein levels (Figure 1G), further suggesting that SAHA does not act through the autophagic pathways to reduce HIF-1α expression.

Taken together the above data suggests that the suppressive effect of SAHA on HIF-1α protein levels is not increasing the activity of HIF-1α clearance pathways.

### SAHA decreases HIF-1α protein expression through HDAC9 inhibition

**Figure 2:**
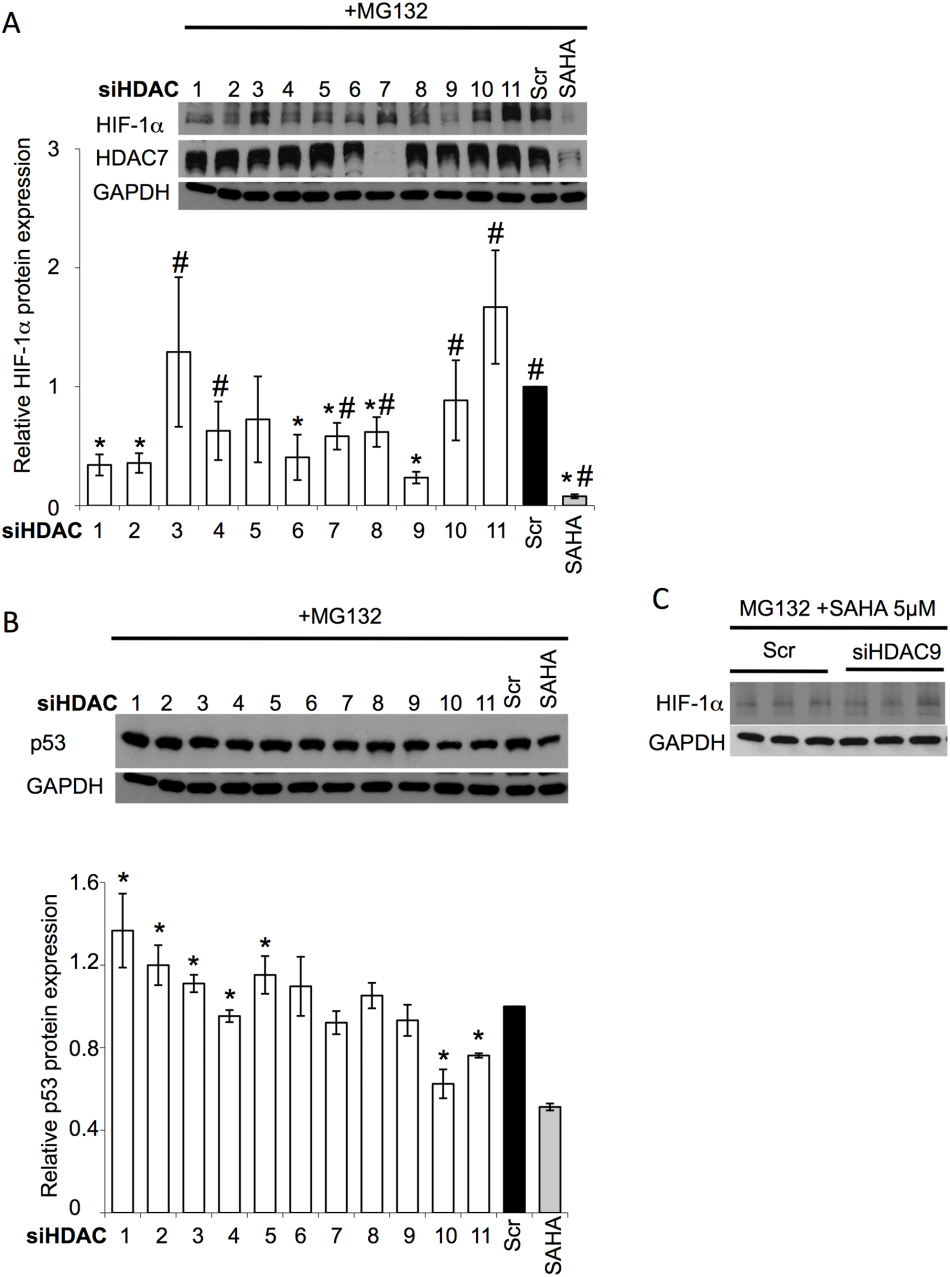
Silencing of HDAC9 represses HIF-1α induction. (**A**) Immunoblot analysis of HIF-1α, HDAC7 and GAPDH protein expression in cell lysates following siRNA-mediated silencing of HDACs 1–11 in HuH7 cells. Quantitative analysis (lower) of the level of HIF-1α in response to silencing of the indicated HDAC in HuH7 cells. Data shown denote the fold change in HIF-1α protein expression relative to scramble (Scr) control (black bar) (mean ± SD, n = 3). Asterisks indicates p<0.05 as determined by two-tailed t-test using Scr control (black bar) as the reference and # indicates p<0.05 as determined by two-tailed t-test using HDAC9 siRNA as the reference. (**B**) Immunoblot analysis of p53 and GAPDH protein expression in cell lysates following siRNA-mediated silencing of HDACs 1–11 in HuH7 cells. Quantitative analysis (lower) of the level of p53 in response to silencing of the indicated HDAC in HuH7 cells. Data shown denote the fold change in p53 protein expression relative to scramble (Scr) control (black bar) (mean ± SD, n = 3). Asterisks indicates p<0.05 as determined by two-tailed t-test using Scr control as the reference. (**C**) Immunoblot analysis of HIF-1α and GAPDH protein expression in cell lysates following siRNA-mediated silencing of HDAC9 (siHDAC9) in the presence of 5 µM SAHA+50 µM MG132 in HuH7 cells. In all panels GAPDH is used as loading control and HDAC7 is used as control to SAHA treatment.

Previous reports have shown that HDAC4 and HDAC6 co-immunoprecipitate with HIF-1α and their inhibition by the HDACi, TSA and LAQ824, compromised the stability and transcriptional activity of HIF-1α [33], [34], [38]. To address the role of individual HDACs in the SAHA-mediated reduction of HIF-1α protein levels, we used siRNA-mediated silencing of individual HDAC family members in HuH7 cells (Figure 2A). There are 18 human HDACs that cluster into five classes by sequence homology [39], [40]: the Zn2+-dependent class I (HDACs 1, 2, 3 and 8), class IIa (HDACs 4, 5, 7 and 9), class IIb (HDACs 6 and 10) and class IV (HDAC11) enzymes, and the NAD+-dependent class III enzymes (sirtuins). The latter are not inhibited by SAHA [41] and are therefore not examined herein. Following an analysis of the HIF-1α protein levels in response to MG132 treatment combined with the siRNA-mediated silencing of HDACs 1–11, we noted that the silencing of HDAC9 (Figure S1A) exhibited the most pronounced reduction of the MG132-induced HIF-1α up-regulation (Figure 2A), resulting in a 4.3-fold decrease in cellular HIF-1α protein levels relative to control (siScramble (siScr) + MG132). We observed that the silencing of HDAC9 also resulted in a reduction of the DFO-induced HIF-1α up-regulation (Figure S1B). The silencing of numerous other HDACs also led to a partial reduction in HIF-1α (Figure 2A), with HDACs 1, 2 and 6 having the most notable effect. These data suggest that the inhibition of several HDACs could explain the robust SAHA-mediated reduction in HIF-1α protein levels, which failed to be recapitulated by any individual siHDAC. Interestingly, the silencing of HDAC10 (Figure S1A) resulted in the most efficient reduction of p53 (Figure 2B), exhibiting a 25% decrease in protein expression, suggesting that the observed SAHA-mediated reduction of HIF-1α and p53 is occurring through inhibition of different HDAC family members.

To provide additional support for our interpretation that the observed SAHA-mediated reduction in HIF-1α protein levels is acting primarily through inhibition of HDAC9, we compared the effect of SAHA alone (Scr - Figure 2C) or in combination with siHDAC9 on HIF-1α protein levels (Figure 2C). The SAHA+siHDAC9 combination did not result in a further reduction of HIF-1α protein levels compared to SAHA alone (Figure 2C), supporting the conclusion that the observed SAHA-mediated HIF-1α repression is acting primarily through a HDAC9-dependent mechanism.

### SAHA and HDAC9 silencing do not affect transcription of HIF-1α

**Figure 3:**
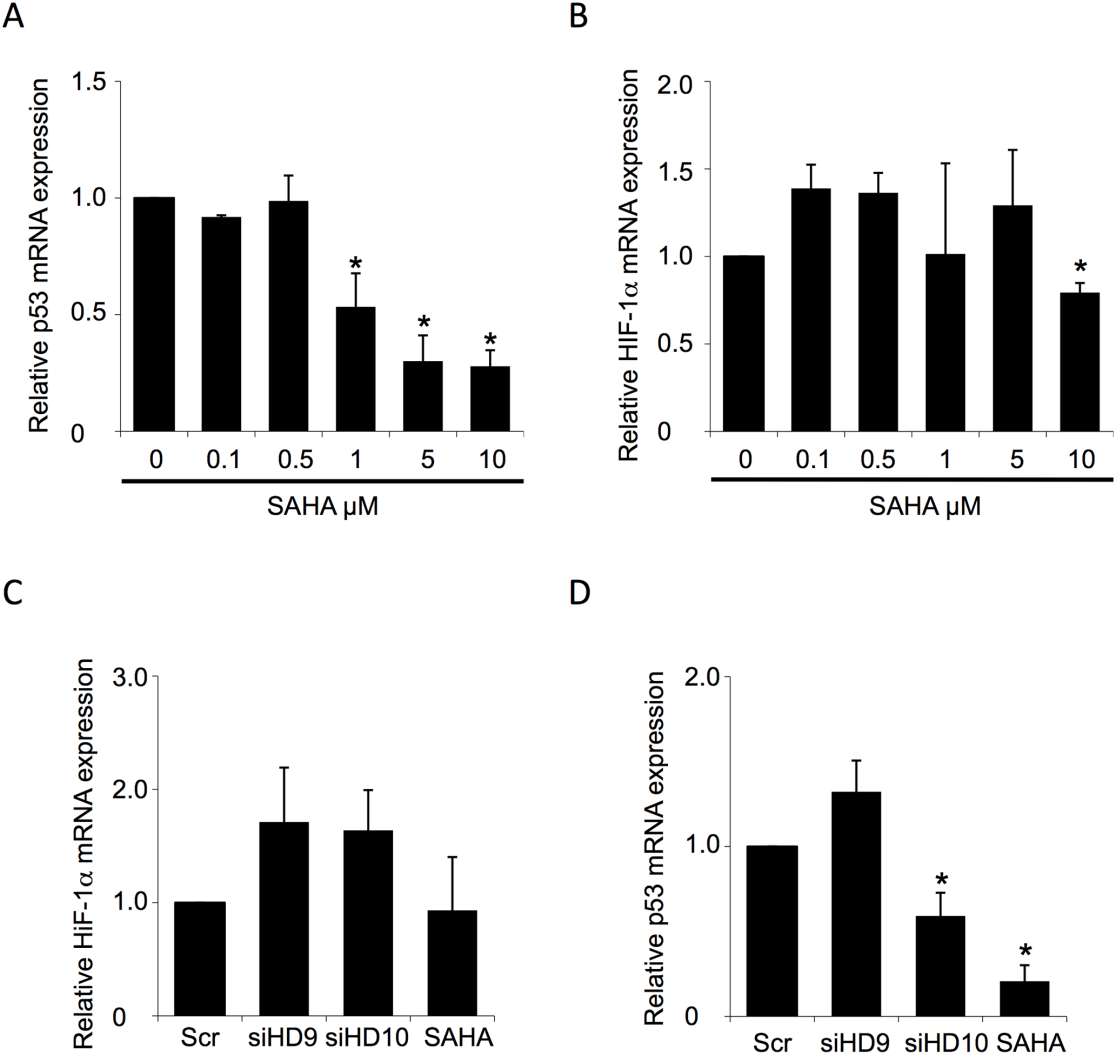
SAHA did not affect the level of HIF-1α mRNA. (**A and D**) qRT-PCR analysis of p53 mRNA level in HuH7 cell line following the indicated concentration of SAHA (**A**) or HDAC9 and HDAC10 silencing (**D**). Data is shown as the fold change of the ratio of p53 to GAPDH mRNA relative to that seen for DMSO (0 µM) (**A**) or scrambled (Scr) siRNA control (**D**) (mean ± SD, n = 3). In all panels, asterisk indicates p<0.05, as determined by two-tailed t-test using scrambled siRNA (Scr) (**D**) or DMSO (0 mM) (**A**) as the reference. (**B–C**) qRT-PCR analysis of HIF-1α mRNA level in HuH7 cell line following the indicated concentration of SAHA (**B**) or HDAC9 and HDAC10 silencing (**C**). Data is shown as the fold change of the ratio of HIF-1α to GAPDH mRNA relative to that seen for DMSO (0 µM) (**B**) or scrambled (Scr) siRNA control (**C**) (mean ± SD, n = 3). In all panels, asterisk indicates p<0.05, as determined by two-tailed t-test using scrambled siRNA (Scr) (**C**) or DMSO (0 µM) (**B**) as the reference.

Previous reports have shown that SAHA treatment can lead to a reduction in p53 mRNA levels in human keratinocyte and colorectal adenocarcinoma cell lines [37], a result consistent with the role of HDACs in epigenetic regulation. To determine whether the level of HIF-1α mRNA is also regulated by SAHA, we measured the levels of HIF-1α and p53 mRNA using real time quantitative PCR (qRT-PCR). Consistent with the previous report discussed above, we observed a dose dependent reduction in p53 mRNA levels in response to SAHA treatment (Figure 3A). Conversely, we did not observe any SAHA-mediated changes in HIF-1α mRNA levels (Figure 3B) at the 5 µM dose where the maximal HIF1α protein reduction is observed, suggesting that transcriptional changes are not responsible for the SAHA-mediated reduction in HIF-1α protein levels.

In light of our observations described above, that the SAHA-mediated effect on HIF-1α and p53 are in part mediated by inhibition of HDAC9 and HDAC10 respectively, we analyzed whether their silencing altered the mRNA levels of HIF-1α or p53. Consistent with the observations reported above for SAHA treatment, the silencing of HDAC9 (Figure S1A) did not alter the cellular mRNA levels of HIF-1α (Figure 3C). Conversely, we did observe that the silencing of HDAC10 led to a significant reduction of p53 mRNA levels (Figure 3D), consistent with the conclusion that the SAHA-mediated reduction in p53 is acting in part through inhibition of HDAC10. Since we did not see any SAHA-mediated changes in HIF-1α transcription or protein degradation, we hypothesized that the SAHA-mediated reduction in cellular levels of HIF-1α could be acting through translational regulation.

### SAHA decreases HIF-1α protein levels by an eIF3G-dependent translation mechanism

Cap-dependent translation is initiated and regulated by the eukaryote Initiation Factor (eIF) family. Recent work has shed light on the role of some of these family members in regulating HIF-1α translation, including eIF2α [42], [43], eIF3E [44] and eIF4E [45]–[47]. It has been shown that the mammalian Target Of Rapamycin Complex 1 (mTORC1) signalling pathway regulates HIF-1α translation by mediating the phosphorylation of eIF4E binding protein 1 (4E-BP1) [45]–[47], which disrupts its binding to eIF4E thereby stimulating cap-dependent translation [48], [49]. Despite this latter observation, we observed a statistically significant decrease in HIF-1α protein expression when we combined mTOR silencing with the MG132 and SAHA treatments (Figure S2A), ruling out a role for mTORC1 in the SAHA-mediated reduction in cellular HIF-1α protein levels.

**Figure 4:**
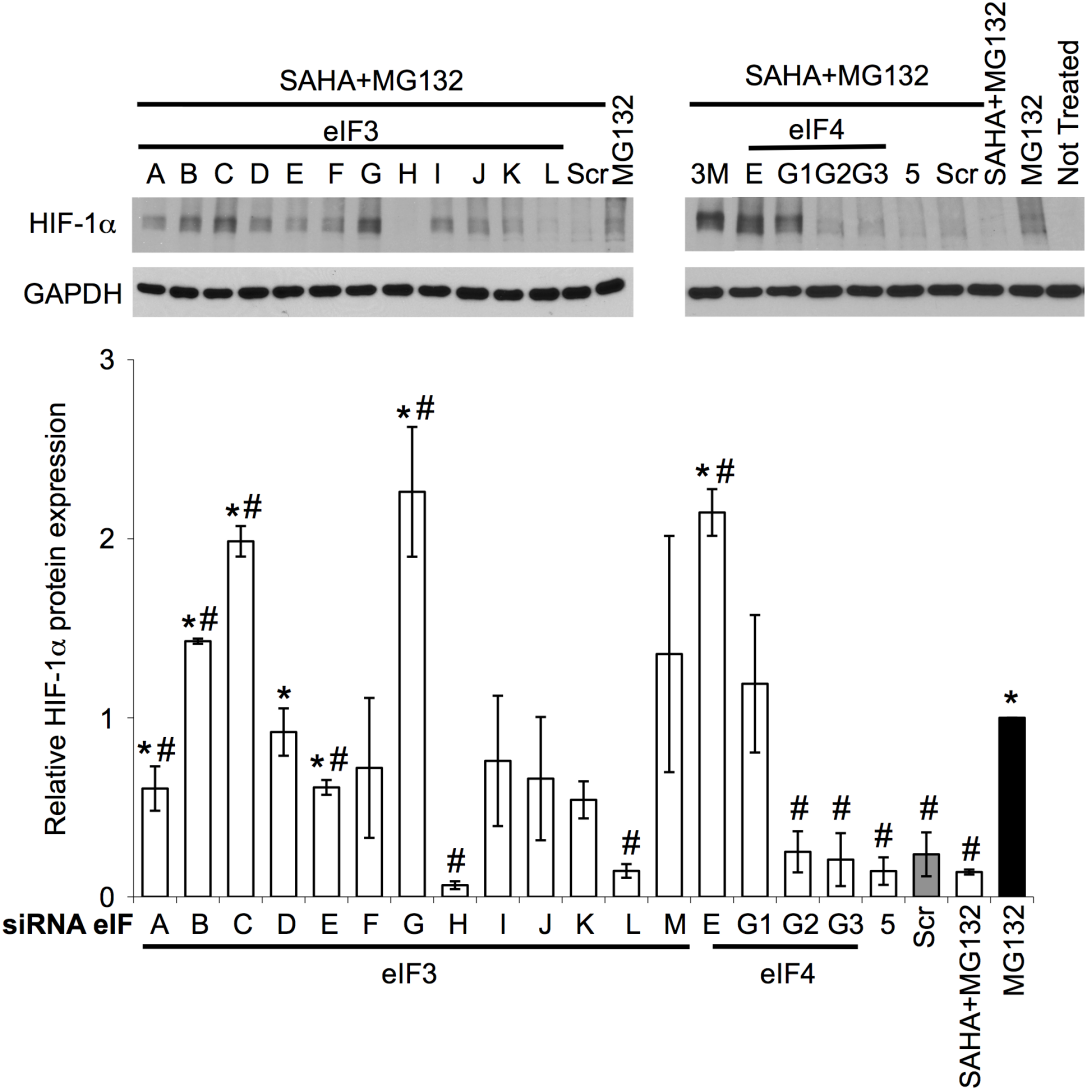
eIF3G silencing reversed SAHA effect on HIF-1α repression in response to hypoxic mimic. Immunoblot analysis of HIF-1α and GAPDH protein expression in cell lysates following siRNA-mediated silencing of eIF3 A-M, eIF4 E, G1–3 and eIF5 in HuH7 cells in presence or absence of SAHA+MG132. Quantitative analysis of the level of HIF-1α in response to silencing of the indicated eIF in HuH7 cells. Data shown denote the fold change in HIF-1α protein expression relative to MG132 treatment alone (black bar) (mean ± SD, n = 3). Asterisks indicates p<0.05 as determined by two-tailed t-test using Scr control (grey bar) as the reference and # indicates p<0.05 as determined by two-tailed t-test using MG132 (black bar) as the reference. In all panels GAPDH is used as loading control.

To address the role of eIF family members in the SAHA-mediated suppression of HIF-1α, we examined the effect of siRNA-mediated silencing of individual eIFs in HuH7 cells following treatment with SAHA and MG132 (Figure 4 and S2B). eIF2 is a heterotrimer composed of α, β and γ subunits where the silencing of the α and γ subunits result in toxicity in HuH7 cells. We therefore targeted the eIF2β subunit to monitor the role of eIF2. We observed that the silencing of eIF3 subunits B, C, G and M, eIF4 subunits E and G1 as well as eIF2β blocked the SAHA-mediated decrease in HIF-1α protein expression, with sieIF3G and sieIF2β having the most pronounced effect (Figure 4 and S2B). We noted a significant increase in toxicity in response to eIF2β silencing, an effect not observed with optimal SAHA dosing, therefore we focused our analysis on the contribution of eIF3G in the SAHA-mediated decrease of HIF-1α. The silencing of eIF3G was also able to block the SAHA-mediated reduction of HIF1α, restoring a partial DFO-induced up-regulation of HIF1α (Figure S2C). We also noted that the silencing of eIF3A, D, E, F, I, J and K were able to partially block the SAHA-mediated decrease in HIF-1α (Figure 4), suggesting a central role for the eIF3 machinery in mediating the action of SAHA on HIF-1α expression.

**Figure 5:**
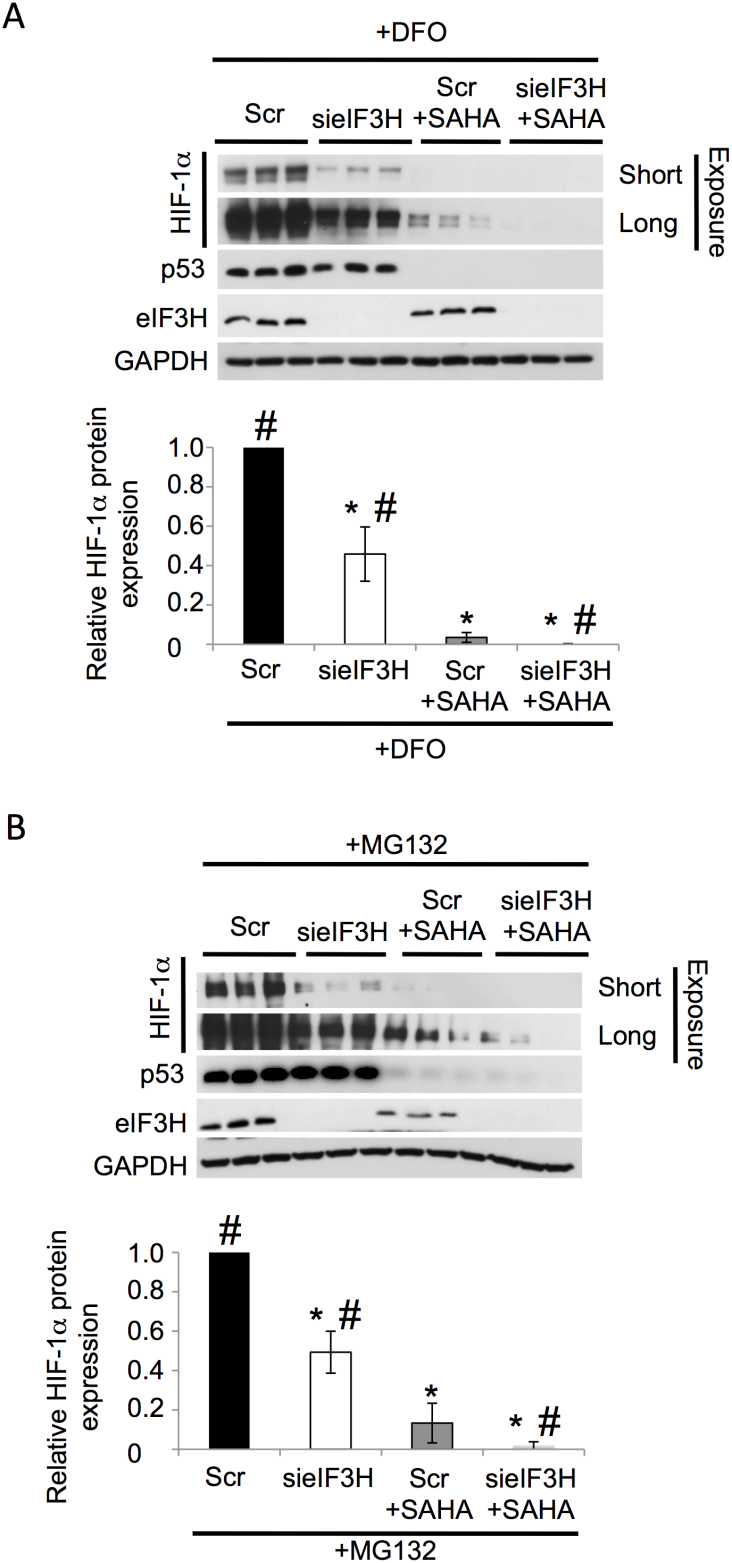
Combined effect of eIF3H silencing and SAHA treament. (**A–B**) Immunoblot analysis of HIF-1α, p53, eIF3H and GAPDH protein expression in cell lysates following siRNA-mediated silencing of eIF3H (sieIF3H) in presence or absence of SAHA+DFO (**A**) or SAHA+MG132 (**B**) in HuH7 cells. Quantitative analysis of the level of HIF-1α in response to silencing of the indicated eIF3H in HuH7 cells. Data shown denote the fold change in HIF-1α protein expression relative to DFO treatment+Scr control (**A**) MG132 treatment+Scr control (**B**) (black bar) (mean ± SD, n = 3). Asterisks indicates p<0.05 as determined by two-tailed t-test using Scr control+DFO (**A**) or Scr control+MG132 (**B**) (black bar) as the reference and # indicates p<0.05 as determined by two-tailed t-test using SAHA+MG132 (**B**) or SAHA+DFO (grey bar) as the reference. In all panels GAPDH is used as loading control.

Conversely, we observed that the silencing of eIF3H in combination with SAHA was able to further decrease HIF-1α protein levels (Figure 4). To further address the role of eIF3H in the SAHA mediated decrease in HIF-1α expression, we examined the effect of eIF3H silencing (sieIF3H) on HIF-1α expression (Figure 5A–B). We noted that the silencing of eIF3H alone was able to reverse the DFO- and MG132-mediated HIF-1α up-regulation (Figure 5A–B), resulting in a ∼2-fold decrease in cellular HIF-1α protein levels relative to control (siScramble (siScr) + MG132 or DFO). Moreover, when eIF3H-silenced cells were treated with SAHA, we observed a further reduction in HIF-1α expression relative to that seen with either treatment alone (Figure 5A–B), suggesting that SAHA and sieIF3H target HIF-1α via parallel pathways.

We also observed that the silencing of eIFs were additive to the SAHA-mediated decrease in p53 expression (Figure S3), a result consistent with our data above that SAHA mediates a reduction in p53 mRNA level and that inhibition of the translational machinery provides a second level of regulation to reduce the expression of p53. These results support the conclusion that the translational machinery in general and eIF3G specifically provide a point of regulation for the HDACi, SAHA in regulating the expression of HIF-1α.

### SAHA does not regulate eIF3G directly

Given that the silencing of eIF3G can inhibit the SAHA-mediated decrease in HIF-1α protein levels, we sought to address its mechanism of action. We first addressed whether eIF3G is directly affected by SAHA treatment. Our analysis revealed that SAHA did not alter the subcellular localization of eIF3G or its protein expression level nor did it change its acetylation state (Figure S4A–C), suggesting that the SAHA-mediated effect on HIF-1α translation is not the result of directly targeting eIF3G. Next, we addressed whether SAHA altered the assembly of the eIF3 complex or its association with the ribosome. Our analysis revealed that SAHA did not alter the interaction of eIF3G with eIF3B or ribosomal protein S6 (Figure S4D) [50].

The eIF3 family activates a number of biological pathways, including the formation of stress granules, which are also regulated by HDACs [51]. Stress granules contain non-translating mRNAs, translation initiation components, and many additional proteins affecting mRNA translation [52]. To explore the possible role of stress granules in the observed effect of SAHA on HIF-1α, we performed an immunofluorescence analysis of the stress granule associated proteins, TIA-1 (T-cell intracellular antigen-1) and TIAR (TIA-1 related protein) [53]. Neither SAHA (Figure S4E) nor SAHA+MG132 (data not shown) treatments induced the formation of stress granules (Figure S4E), a phenomenon that was observed in response to Tunicamycin treatment (positive control) (Figure S4E). These data suggest that changes in stress granules formation or regulation are not involved in the SAHA-mediated changes in HIF-1α protein levels.

Although we noted that the silencing eIF3G alone did not impact the expression level of HIF-1α (Figure S5A), the overexpression of Myc-tagged eIF3G, in the absence of SAHA treatment, induced a statistically significant increase in HIF-1α and p53 protein levels (Figure S5B). Combining the overexpression of eIF3G with the SAHA treatment did not further alter the SAHA-mediated reduction in HIF-1α, further supporting the conclusion that eIF3G is required for the SAHA-mediated impediment of HIF-1α translation.

## Discussion

HIF-1α is a master regulator in angiogenesis, metabolic reprogramming, epithelial-mesenchymal transition, stem cell maintenance, invasion, metastasis and resistance to several therapies making it a major target for therapeutic development in cancer [18], [19], however development of small-molecule HIF-1α inhibitors has been a major challenge. The HDACi, SAHA, has been reported to provide therapeutic benefit in a number of oncogenic conditions, including pancreatic, breast, prostate, colon, liver and hematological cancers. SAHA is well tolerated when administrated either intravenously or orally, and has been found to have antitumor activity in advanced solid tumors refractory to other therapeutic interventions. By consequence, SAHA represents a promising anti-tumor drug in both preclinical and clinical development. Interestingly, HDACi have been reported to be potent HIF-1α inhibitors [16], [17], however, the mechanism of action for this inhibition remains to be elucidated. Here, we found that SAHA was able to repress the expression of the angiogenic factor, HIF-1α without altering its transcription or degradation. Rather we report that the HDAC inhibitor, SAHA, regulates HIF-1α translation in HCC cell lines, in a mechanism dependent on the eukaryotic translation initiation machinery.

Under normoxic conditions and in the absence of growth factor stimulation, the HIF-1α protein is targeted for degradation [23], [24]. The accumulation of HIF-1α can therefore be altered through increased protein synthesis or inhibition of protein degradation. Herein, we observe that SAHA can reverse the aberrant accumulation of HIF-1α seen in response to inhibition of HIF-1α degradation such as treatment with the prolyl hydroxylase inhibitors, CoCl_2_, desferrioxamine (DFO) and dimethyloxallyl glycine (DMOG) or the proteasome inhibitor, MG132. Additionally, SAHA mediates a transcriptional block of p53, which would be predicted to reduce the MDM2-mediated ubiquitination and subsequent proteosomal degradation of HIF-1α [21], [25], [26]. These data clearly demonstrate that the SAHA effect on HIF-1α is occurring at the level of protein expression rather than by promoting the degradation of this angiogenic factor, however no changes in HIF-1α mRNA levels were observed in response to SAHA treatment.

The role of SAHA in protein translation has already been demonstrated for other proteins. Indeed, SAHA has been shown to exhibit a regulatory function on the mTOR pathway to suppress the translation of cyclin D1 [56], a pathway previously shown to also regulate HIF-1α translation [45]–[47]. Additionally, Sorafenib, the only drug clinically approved for patients with advanced HCC, has been shown to induce suppression of mTOR/p70S6K/4E-BP1 pathway [54], which could account for its therapeutic benefit in HCC. However, we failed to detect a dependence on the mTOR pathway for the SAHA-mediated reduction in HIF-1α (Figure S2A). These data could account for the synergistic effect of the combined SAHA and Sorafenib treatment currently under clinical investigation for a number of cancers, including HCC.

More generally, protein translation is subject to a number or regulatory steps, the first of which involves the eukaryotic initiation factors (eIF), which are involved in mRNA cap binding, ribosome recruitment and start codon scanning. Here the eIF4F complex, which is composed of the cap binding protein eIF4E, the ATP-dependent RNA helicase, eIF4A and the scaffolding protein eIF4G1, binds to the mRNA molecule and recruits the eIF3-bound ribosome through interaction with eIF4G1. The eIF3 complex is composed of 13 subunits (A through M) with eIF3A, B, C, E, F & H composing the essential core of the initiation complex [55] and the remaining subunits providing poorly defined regulatory functions. Herein, we report a clear dependence on the presence of a functional translational initiation complex for the SAHA-mediated reduction in HIF-1α protein level, with the eIF4E, 4G1 and 3G subunits providing the most dramatic block of the SAHA-mediated inhibition of HIF-1α. Unlike eIF4E and 4G1, which are critical components of translation initiation, eIF3G has been shown to provide a more regulatory role [55] by promoting mRNA circularization [56]. Nonetheless, these data provide evidence that the impact of SAHA on HIF-1α expression is occurring at the level of translational regulation. Furthermore, our data shows that these factors must be present at endogenous expression levels for the SAHA-mediated effect be observed and that their silencing mediate sustained or increased HIF-1α expression.

The translation initiation factor eIF3G has never been demonstrated to play an important role in tumor development. Here, our results further support the conclusion that eIF3G is a target for the anti-cancer properties of SAHA for the treatment of HCC and possibly other types of cancers and that its therapeutic benefit would be independent of a potential increased expression level of eIF3G (Figure S5B). Additionally, increased expression of eIF3 subunits have been reported in cancer [51]. Among those subunits, the overexpression of eIF3H has been found to be increased in several types of cancer [57], [58], where it has been speculated to regulate translation of mRNAs specifically involved in cell growth or apoptosis [59] and consequently may favor oncogenic transformation. Our results are in agreement with these ideas and suggest an eIF3H-mediated control of HIF-1α translation that could explain in part its oncogenic properties. Interestingly, combining eIF3H silencing with SAHA treatment had an additive effect on HIF-1α repression. This observation could open a new avenue in therapeutic development in cancer by screening for small molecule inhibitors of eIF3H activity or expression.

Our characterization of the mechanism of action for the SAHA-mediated HIF-1α protein repression indicates that SAHA acts through HDAC9 silencing. Previous reports have highlighted the role of HDAC family members, namely 1, 3, 4, 6 and 7, on HIF-1α protein stability and activity [34; 35; 36; 53;5 4]. However, only the silencing of HDAC4 and 6 were shown to regulate the protein expression level of HIF-1α, by altering its post-translational acetylation and its subsequent interaction with the Hsp70/Hsp90 chaperone machinery [34]; [35]; [36]. Our experimental data demonstrates that HDAC9 is a prominent target for the SAHA-mediated reduction in HIF-1α protein expression. Recently, a study using high-resolution copy-number analysis and whole-exome sequencing of HCC tumors [60] identified 135 homozygous deletions and 994 somatic mutations of genes with predicted functional consequences, including a missense mutation in the HDAC9 gene (Leu1043Gln) [60]. The functional consequences of this mutation are unclear, but in light of our recent data, a deregulation of HDAC9 activity could impact the expression and function of HIF-1α and possibly other oncogenes. Furthermore, there are 9 variants of HDAC9 differing in their 3′, 5′ UTR regions as well as in the coding sequence. In light of this information, future studies addressing which variants of HDAC9 are targeted by SAHA to account for the reduction in HIF-1α protein expression are warranted.

**Figure 6:**
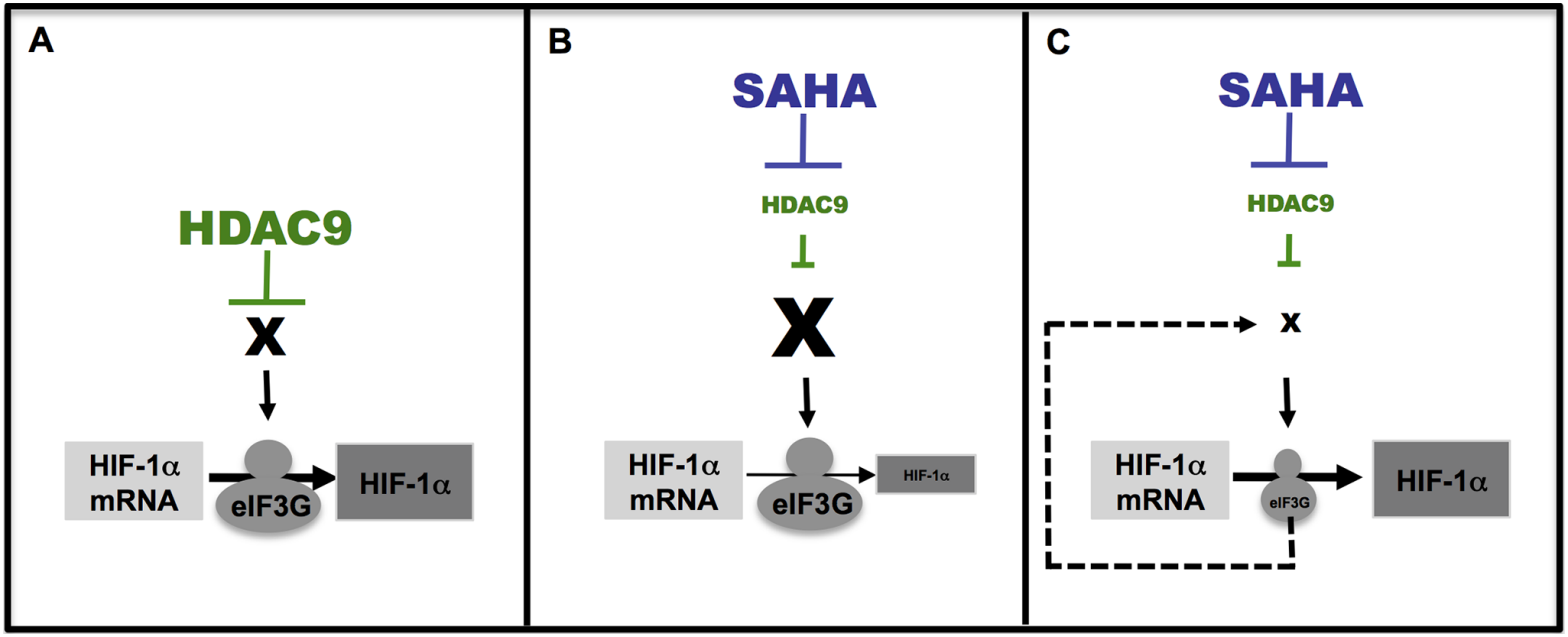
A model for SAHA mediated effects on HIF-1α translation. In non-treated conditions (left panel-A), HIF-1α translation is controlled by HDAC9 and then HIF-1α mRNA is translated in protein. Following SAHA treatment (middle panel-B), HDAC9 is inhibited and we suggest that SAHA could promote mRNA and protein expression of an unknown protein (referred as X) that controls HIF-1α translation. As a result of this, the unknown protein could repress specifically HIF-1α translation. Upon the combined treatment SAHA and eIF3G silencing (right panel-C), we suggest that eIF3G may play a regulatory role in translation of the mRNA coding the unknown protein. By consequence, the silencing of eIF3G results in the inhibition of the unknown protein translation. As the expression of this unknown protein is down regulated, HIF-1α translation is not repressed anymore and could be translated *de* *novo*.

Our combined results lead us to suggest a model where in absence of treatment, HIF-1α translation is enhanced by HDAC9 activity (Left panel-Figure 6). In this condition, HIF-1α translation is regulated by cellular oxygen levels, being either degraded or imported to the nucleus (Left panel-Figure 6A). Upon SAHA treatment, HDAC9 is inhibited and HIF-1α translation is repressed (Middle panel-Figure 6B). SAHA may induce the expression of other, yet identified, key protein(s) (referred as X in Figure 6) that negatively modulate HIF-1α translation. Upon eIF3G silencing, these other HIF-1α modulators are differentially expressed thereby blocking the SAHA-mediated repression of HIF-1α (Right panel-Figure 6C).

In summary, we report that the HDAC inhibitor, SAHA, regulates HIF-1α translation in HCC cell lines, in a mechanism dependent on the eukaryotic translation initiation machinery. Our results highlight the importance of further investigating the functional relationship between eIFs and HIF-1α for the possible therapeutic development of anti-cancer compounds.

## Materials and Methods

### Materials

HDACi (suberoylanilide hydroxamic acid; SAHA) was purchased from Cayman Chemical (Ann Arbor, MI, USA). Ammonium chloride, cobalt chloride (II), desferrioxamine (DFO), dimethyloxallyl glycine (DMOG) and MG132 (Z-leu-leu-leu-al) were purchased from Sigma (StLouis, MO, USA). Tunicamycin was purchased from Calbiochem (Merck Bioscience, Darmstadt, Germany) and was dissolved in DMSO to a final stock concentration of 10 mg/ml. SiRNA were obtained from Ambion (Austin, TX, USA). RNA extraction kits were from Qiagen (Valencia, CA, USA). Rabbit anti-human HDAC7 and anti-Myc tag antibodies were purchased from Abcam (Cambridge, MA, USA). Anti-human H3 acetylated antibody was purchased from Millipore (Billerica, MA, USA). Rabbit anti-human eIF3G antibody was purchased from Novus Biologicals (Littleton, CO, USA). Mouse anti-human Hsp90, TIA-1/TIAR and goat anti-human eIF3B antibodies were purchased from Santa Cruz Biotechnology (Santa Cruz, CA, USA). Mouse anti-human GAPDH was purchased from Genetex (Irvine, CA, USA). Mouse anti-human HIF-1α antibody was purchased from BD biosciences (Franklin Lakes, NJ, USA). Rabbit anti-human LC3 antibody was purchased from Sigma (St Louis, MO, USA). Mouse anti-human p53 was purchased from Calbiochem (Merck Bioscience, Darmstadt, Germany). Rabbit anti-human Total acetyl antibody, rabbit anti-human eIF3H and mouse anti-human ribosomal protein S6 antibody were purchased from Cell Signaling (Danvers, MA, USA).

### DNA Constructs

eIF3G cDNA was cloned from human liver cDNA using the Gateway technology (Invitrogen-Carlsbad, CA, USA) in pRK5-Myc expression plasmid. Primers used for cloning are 5′-ggggacaagtttgtacaaaaaagcaggcttgcctactggagactttgattcgaa-3′ and 5′-ggggaccactttgtacaagaaagctgggtattagttggtggacggcttg-3′. eIF3G cDNA devoid of ATG, were amplified by PCR using the Platinium *Taq* DNA Polymerase High Fidelity (Invitrogen- Carlsbad, CA, USA). These PCR products were purified using PCR Clean-Up System Promega (Wisconsin, USA) and recombined into pDONR221 using the Gateway BP clonase (Invitrogen-Carlsbad, CA, USA). These plasmids were then transformed into competent DH5α cells and positive clones selected and sequenced. These clones were then recombined into destination vectors (pRK5-Myc) from Eric Chevet Lab (Bordeaux, France) using LR clonase (Invitrogen-Carlsbad, CA, USA).

### Cell culture, transfections and treatments

HuH7 and Hep3B cells were obtained from Eric Chevet Lab (Bordeaux, France). These cells were cultured in DMEM containing 10% (v/v) fetal bovine serum (FBS). Cells were treated with the indicated concentrations of SAHA, MG132, desferrioxamine (DFO), dimethyloxallyl glycine (DMOG), ammonium chloride, cobalt chloride (II) and tunicamycin or vehicle (DMSO, water) in complete growth media (above). HDACs silencing and eIFs silencing were performed with RNAiMax (Invitrogen, Carlsbad, CA, USA) as per the manufacturer’s protocol using 50 nM of the indicated siRNA. HuH7 cells were transiently transfected with the pRK5-Myc eIF3G plasmid. Transfection were done using PromoFectine-hepatocyte (PromoCell, Heidelberg, Germany) according to the manufacturer’s recommendations.

### siRNA-mediated silencing of HDACs and eIFs

HuH7 cells were plated in 12-wells plates and grown to 30–40% confluence. Silencing of individual HDACs and eIFs were performed as indicated above and as described previously [35].

### qRT-PCR

Quantitative PCR was performed as described previously [61] and using the following primers and using the following primers for HIF-1α: 5′-gaacaaaacacacagcgaag-3′ and 5′-acaaatcagcaccaagcag-3′; p53: 5′-tctccacttcttgttcccc-3′ and 5′-ctccccacaacaaaacacc-3′; qRT-PCR for HDAC9 and HDAC10 were performed as described previously [35]. RNA was standardized by quantification of GAPDH mRNA using primers 5′-AAGGTGAAGGTCGGAGTCAA-3′ and 5′-CATGGGTGGAATCATATTGG-3′, and all values are expressed relative to GAPDH.

### Immunoblotting

Cell lysates were prepared in 50 mM Tris-HCl, 150 mM NaCl, 1% (v/v) Triton X-100 and protease inhibitors and protein concentrations determined by Bradford protein assay (Thermo Scientific, Rockford, IL, USA). Total protein were resuspended in 1X SDS sample buffer containing DTT and incubated at 95°C for 5 min. The samples 10–25 µg of total protein are then separated on a 10% (v/v) SDS-PAGE, transferred to nitrocellulose and immunoblotted with anti-human HIF-1α antibody (BD biosciences, Franklin Lakes, NJ, USA) or indicated primary antibodies. Detection was performed using chemiluminescence and the appropriate horseradish peroxidase (HRP)-conjugated secondary antibodies.

### Immunoprecipitation

Cell lysates were prepared as described in Immunoblotting section and 1 mg of pre-cleared (1 h at 4°C with Protein G Sepharose) cell lysate was incubated overnight at 4°C with the indicated antibody (anti-Myc tag). Immunoprecipitations with non-coupled antibodies were incubated for 1 h at 4°C with Protein G Sepharose beads. Beads and bound proteins are washed with three changes of lysis buffer without protease inhibitor and resuspended in 1X SDS sample buffer containing DTT and incubated at 95°C for 5 min. Input and bound material were separated on a 10% (v/v) SDS-PAGE, transferred to nitrocellulose and immunoblotted with anti-human total acetylated antibody. Detection was performed as described in Immunoblotting section.

### ImmunoFluorescence

For staining of eIF3G protein and stress granules, HuH7 cells were grown in 24-well plates, fixed with 4% paraformaldehyde for 10 min, and permeabilized with 0.1% Triton X-100 for 1 min at room temperature. Cells were then incubated with anti-Myc Tag and anti-TIA-1/TIAR antibodies for 1 h at room temperature followed by incubation with fluorescently labeled secondary antibodies. Protein subcellular localization was performed using an epifluorescence microscope with a Zeiss 63 1.4 oil immersion objective, recorded with a digital camera, and analyzed with Northern Eclipse software (Emprix Imaging, Mississauga, Ontario, Canada). The percentage of stress granules per cells was performed as described previously [62].

## Supporting Information

Figure S1 **Silencing of HDAC9 represses HIF-1α protein expression.** (**A**) qRT-PCR analysis of mRNA levels of HDAC 9 (Left) and 10 (Right) in HuH7 cell line following their respective silencing. Data are shown as the fractional recovery of the indicated HDAC mRNA, normalized to GAPDH mRNA, relative to the level seen with scrambled (Scr) siRNA (mean ± SD, n = 3). (**B**) Immunoblot analysis of HIF-1α, p53 and GAPDH protein expression in cell lysates following siRNA-mediated silencing of HDAC9 or Scr (Scramble control)+100 µM desferrioxamine (DFO) for 18 h in HuH7 cells and following treatment of HuH7 cells with 5 µM SAHA for 24 h+100 µM desferrioxamine (DFO) for 18 h. In all panels GAPDH is used as loading control.
(TIF)

Figure S2 **Effects of eIF2β, eIF3G and mTOR silencing on HIF-1α protein level.** (**A**) Immunoblot analysis of HIF-1α and GAPDH protein expression in cell lysates following siRNA-mediated silencing of mTOR in HuH7 cells in presence of SAHA+MG132 or following Scr (Scramble control) in presence of MG132. Quantitative analysis (lower) of the level of HIF-1α following siRNA-mediated silencing of mTOR in HuH7 cells in presence of SAHA+MG132. Data shown denote the fold change in HIF-1α protein expression relative to scramble (Scr)+MG132 control (black bar) (mean ± SD, n = 6). Asterisks indicates p<0.05 as determined by two-tailed t-test using Scr+MG132 as the reference, # indicates p<0.05 as determined by two-tailed t-test using Scr+SAHA+MG132 as the reference. (**B**) Immunoblot analysis of HIF-1α and GAPDH protein expression in cell lysates following siRNA-mediated silencing of eIF2β in HuH7 cells in presence of SAHA+MG132. (**C**) Immunoblot analysis of HIF-1α, eIF3G and GAPDH protein expression in cell lysates following siRNA-mediated silencing of eIF3G in HuH7 cells in presence of SAHA+DFO or DFO. In all panels GAPDH is used as loading control.
(TIF)

Figure S3 **eIF silencing did not reverse SAHA effect on p53 protein level.** Immunoblot analysis of p53, HDAC7 and GAPDH protein expression in cell lysates following siRNA-mediated silencing of eIF3 A-M, eIF4 E, G1–3 and eIF5 in HuH7 cells in presence or absence of SAHA+MG132. Quantitative analysis (lower) of the level of p53 in response to silencing of the indicated eIF in HuH7 cells. Data shown denote the fold change in p53 protein expression relative to Scrambled (Scr) siRNA control (black bar) (mean ± SD, n = 3). Asterisks indicates p<0.05 as determined by two-tailed t-test using Scr control (black bar) as the reference. In all panels GAPDH is used as loading control and HDAC7 is used as control to SAHA treatment.
(TIF)

Figure S4 **SAHA does not target eIF3G directly.** (**A**) Immunofluorescence analysis of Myc tag protein expression following DMSO and SAHA+MG132. (**B**) Immunoblot analysis of eIF3G, acetylated histone H3 (Acetyl H3) and GAPDH protein expression in cell lysates following DMSO or SAHA treatment in HuH7 cells. (**C**) Immunoblot analysis of eIF3G, acetylated histone H3, acetylated lysine and Hsp90 before (input/upper) and after immunoprecipitation of Myc tag (IP Myc/lower) in HuH7 cells following DMSO or SAHA (5 µM) treatment. (**D**) Immunoblot analysis of Myc-eIF3G, ribosomal protein S6 and eIF3B before (input/left) and after immunoprecipitation of Myc tag (IP Myc/right) in HuH7 cells following DMSO or SAHA (5 µM) treatment. (**E**) Immunofluorescence analysis of TIA-1/TIAR protein expression following DMSO, SAHA (5 µM) or Tunicamycin (2 µg/ml) treatments for 24 h. Quantitative analysis (lower) of TIA-1/TIAR stress granules in HuH7 cells in response to DMSO, SAHA or tunicamycin (TUN) treatments. The percentage of stress granules per cells was obtained as described previously [62]. Data shown denote the fold change in TIA-1/TIAR stress granules relative to DMSO (white bar) treatment (mean ± SD, n = 6). Asterisks indicates p<0.05 as determined by two-tailed t-test using DMSO as the reference. In all panels Hsp90 and GAPDG are used as loading control and acetylated histone H3 is used as control to SAHA treatment.
(TIF)

Figure S5 **eIF3G is required for SAHA-mediated repress of HIF-1α** **translation.** (**A**) Immunoblot analysis of HIF-1α, eIF3G and GAPDH protein expression in cell lysates following siRNA-mediated silencing of eIF3G in HuH7 cells in presence of MG132. (**B**) Immunoblot analysis (left) of HIF-1α, p53, Myc tag, acetylated histone H3 (Acetyl H3) and GAPDH protein expression in cell lysates following Myc tag or Myc-eIF3G overexpression in HuH7 cells in presence or absence of SAHA+MG132. Quantitative analysis (right) of the level of HIF-1α and p53 in response to Myc-eIF3G overexpression in HuH7 cells in presence or absence of SAHA+MG132. Data shown denote the fold change in HIF-1α (black bar) and p53 (grey bar) protein expression relative to Myc tag overexpression (Myc) (mean ± SD, n = 6). Asterisks indicates p<0.05 as determined by two-tailed t-test using Myc tag overexpression in presence of DMSO+MG132 as the reference. In all panels GAPDH is used as loading control and acetylated histone H3 is used as control to SAHA treatment.
(TIF)
