# Supplementary figures and images for: The Histone Deacetylase Inhibitor, Vorinostat, Represses Hypoxia Inducible Factor 1 Alpha Expression through Translational Inhibition
Source: PLoS One. 2014 Aug 28;9(8):e106224. doi: 10.1371/journal.pone.0106224 (PMC4148404; doi:10.1371/journal.pone.0106224)

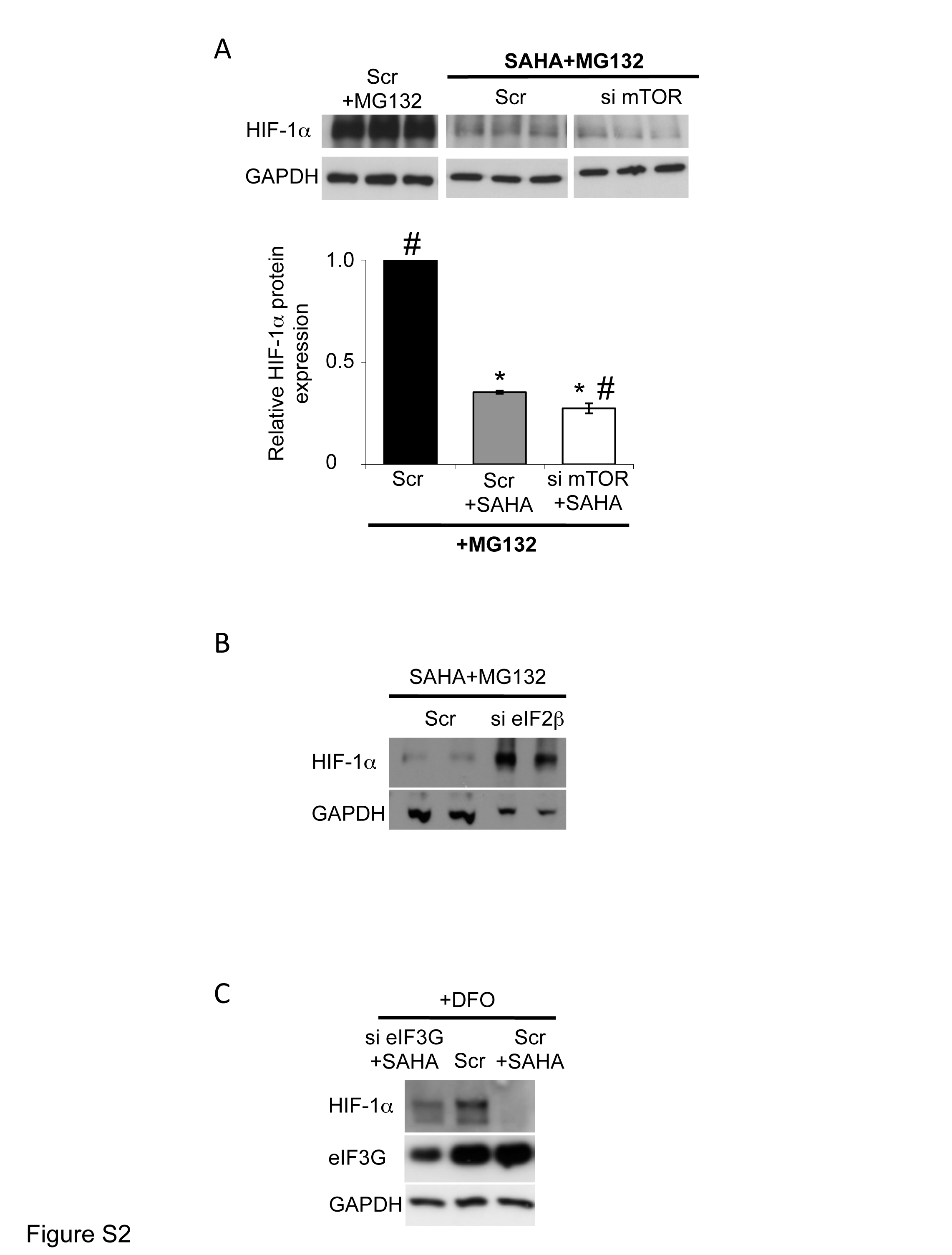

Supplement: Figure S2 — Effects of eIF2β, eIF3G and mTOR silencing on HIF-1α protein level. (A) Immunoblot analysis of HIF-1α and GAPDH protein expression in cell lysates following siRNA-mediated silencing of mTOR in HuH7 cells in presence of SAHA+MG132 or following Scr (Scramble control) in presence of MG132. Quantitative analysis (lower) of the level of HIF-1α following siRNA-mediated silencing of mTOR in HuH7 cells in presence of SAHA+MG132. Data shown denote the fold change in HIF-1α protein expression relative to scramble (Scr)+MG132 control (black bar) (mean ± SD, n = 6). Asterisks indicates p<0.05 as determined by two-tailed t-test using Scr+MG132 as the reference, # indicates p<0.05 as determined by two-tailed t-test using Scr+SAHA+MG132 as the reference. (B) Immunoblot analysis of HIF-1α and GAPDH protein expression in cell lysates following siRNA-mediated silencing of eIF2β in HuH7 cells in presence of SAHA+MG132. (C) Immunoblot analysis of HIF-1α, eIF3G and GAPDH protein expression in cell lysates following siRNA-mediated silencing of eIF3G in HuH7 cells in presence of SAHA+DFO or DFO. In all panels GAPDH is used as loading control. (TIF) [file pone.0106224.s002.tif]

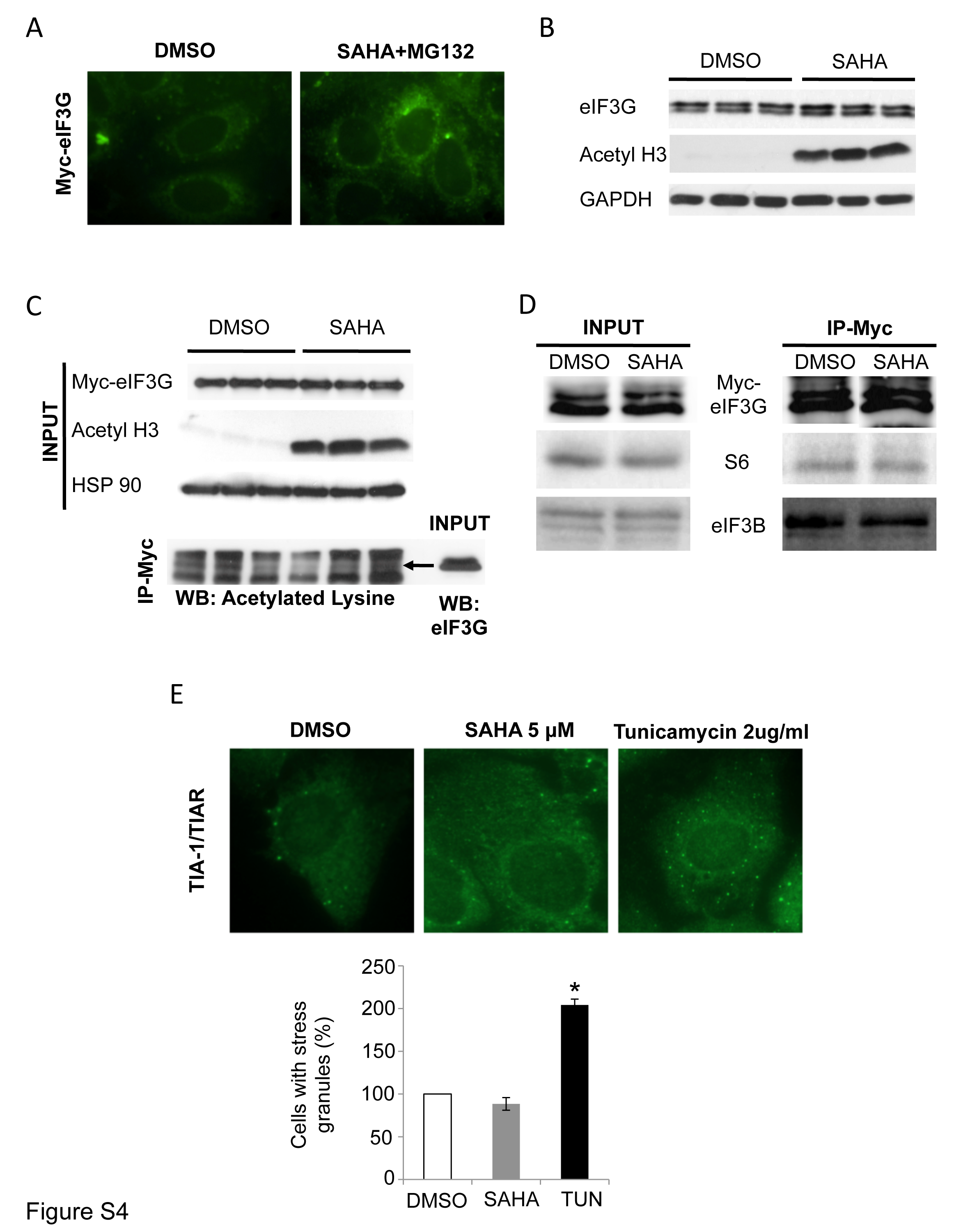

Supplement: Figure S4 — SAHA does not target eIF3G directly. (A) Immunofluorescence analysis of Myc tag protein expression following DMSO and SAHA+MG132. (B) Immunoblot analysis of eIF3G, acetylated histone H3 (Acetyl H3) and GAPDH protein expression in cell lysates following DMSO or SAHA treatment in HuH7 cells. (C) Immunoblot analysis of eIF3G, acetylated histone H3, acetylated lysine and Hsp90 before (input/upper) and after immunoprecipitation of Myc tag (IP Myc/lower) in HuH7 cells following DMSO or SAHA (5 µM) treatment. (D) Immunoblot analysis of Myc-eIF3G, ribosomal protein S6 and eIF3B before (input/left) and after immunoprecipitation of Myc tag (IP Myc/right) in HuH7 cells following DMSO or SAHA (5 µM) treatment. (E) Immunofluorescence analysis of TIA-1/TIAR protein expression following DMSO, SAHA (5 µM) or Tunicamycin (2 µg/ml) treatments for 24 h. Quantitative analysis (lower) of TIA-1/TIAR stress granules in HuH7 cells in response to DMSO, SAHA or tunicamycin (TUN) treatments. The percentage of stress granules per cells was obtained as described previously [62]. Data shown denote the fold change in TIA-1/TIAR stress granules relative to DMSO (white bar) treatment (mean ± SD, n = 6). Asterisks indicates p<0.05 as determined by two-tailed t-test using DMSO as the reference. In all panels Hsp90 and GAPDG are used as loading control and acetylated histone H3 is used as control to SAHA treatment. (TIF) [file pone.0106224.s004.tif]

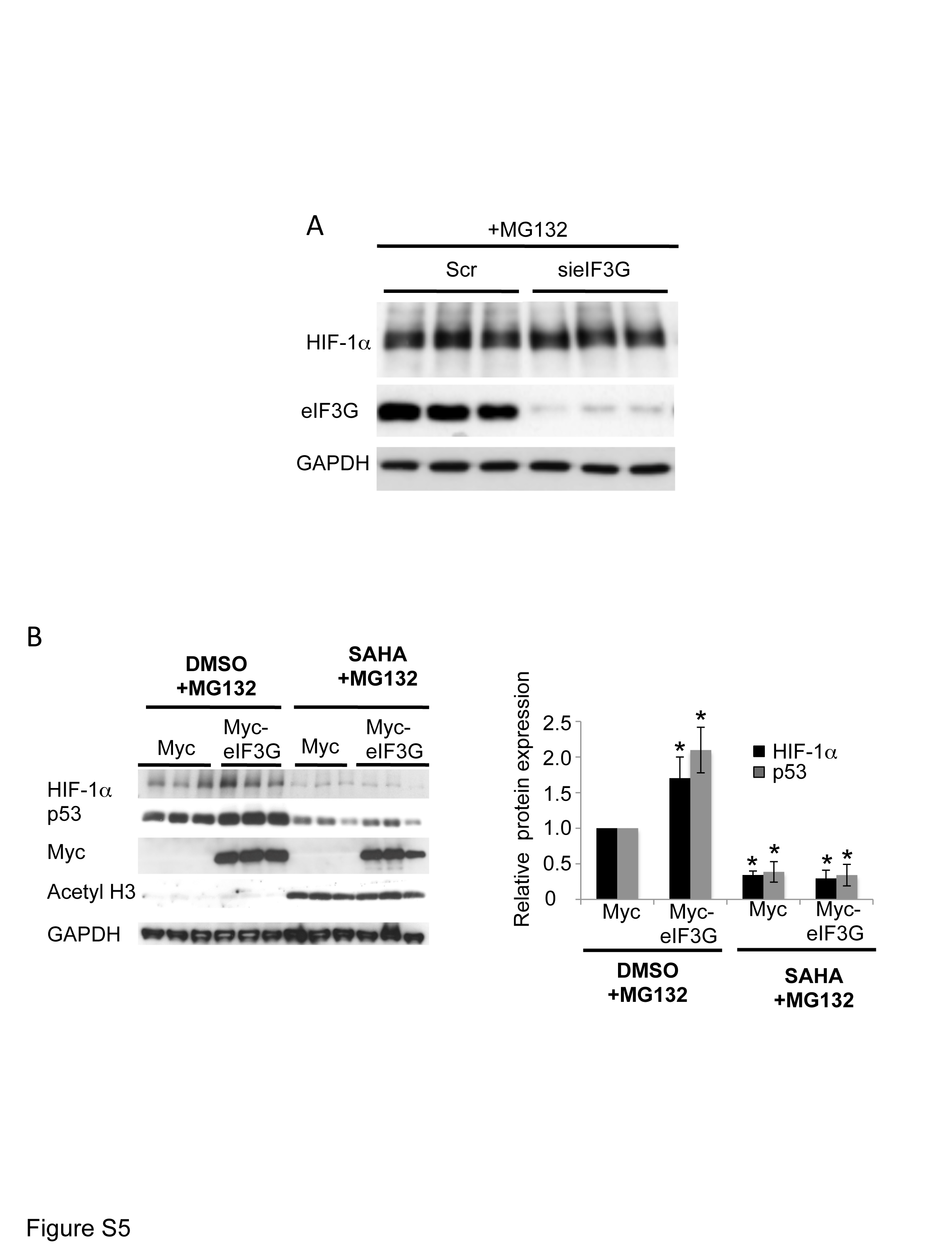

Supplement: Figure S5 — eIF3G is required for SAHA-mediated repress of HIF-1α translation. (A) Immunoblot analysis of HIF-1α, eIF3G and GAPDH protein expression in cell lysates following siRNA-mediated silencing of eIF3G in HuH7 cells in presence of MG132. (B) Immunoblot analysis (left) of HIF-1α, p53, Myc tag, acetylated histone H3 (Acetyl H3) and GAPDH protein expression in cell lysates following Myc tag or Myc-eIF3G overexpression in HuH7 cells in presence or absence of SAHA+MG132. Quantitative analysis (right) of the level of HIF-1α and p53 in response to Myc-eIF3G overexpression in HuH7 cells in presence or absence of SAHA+MG132. Data shown denote the fold change in HIF-1α (black bar) and p53 (grey bar) protein expression relative to Myc tag overexpression (Myc) (mean ± SD, n = 6). Asterisks indicates p<0.05 as determined by two-tailed t-test using Myc tag overexpression in presence of DMSO+MG132 as the reference. In all panels GAPDH is used as loading control and acetylated histone H3 is used as control to SAHA treatment. (TIF) [file pone.0106224.s005.tif]
